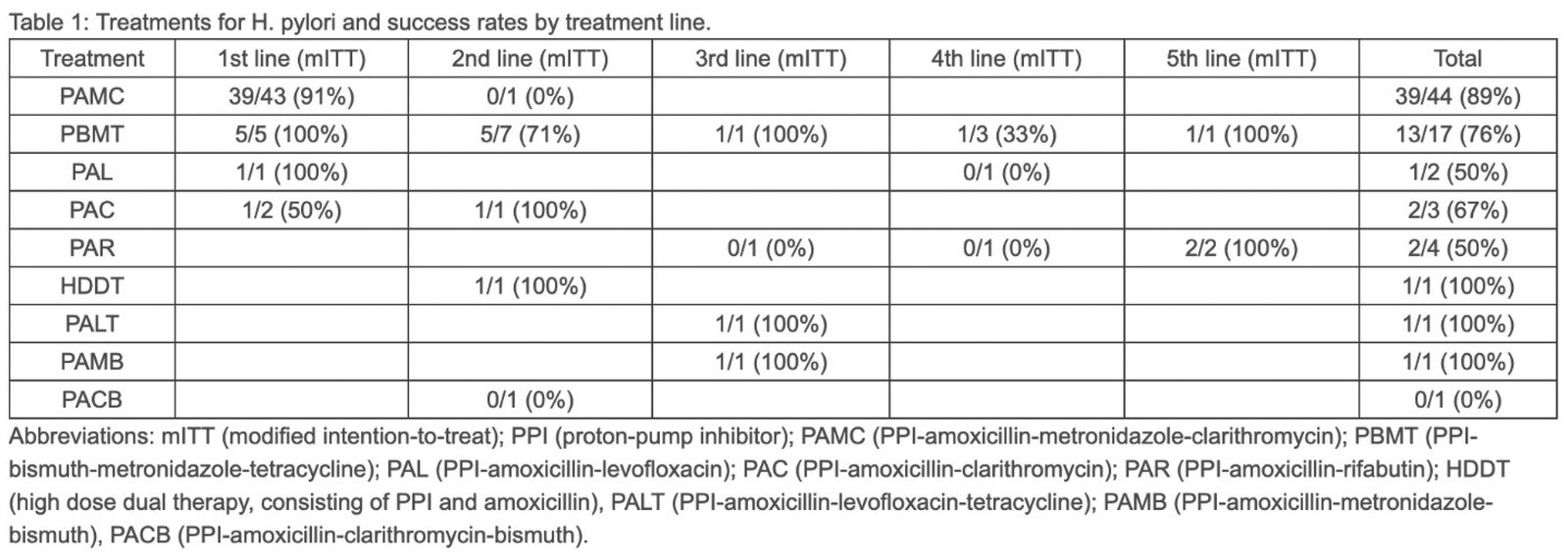# Poster Session II - A188 FIRST PROSPECTIVE CANADIAN DATA OF THE WORLD-WIDE REGISTRY ON *HELICOBACTER PYLORI* MANAGEMENT (WORLDHPREG)

**DOI:** 10.1093/jcag/gwaf042.188

**Published:** 2026-02-13

**Authors:** T Krahn, G Ou, C Rueda-Clausen, M Miles, R Odsen, A Singla, O Farrés, P Parra, L Moreira, O P Nyssen, S Veldhuyzen Van Zanten, J P Gisbert

**Affiliations:** University of Alberta Faculty of Medicine & Dentistry, Edmonton, AB, Canada; The University of British Columbia Faculty of Medicine, Vancouver, BC, Canada; University of Saskatchewan, Regina, SK, Canada; Dalhousie University Faculty of Medicine, Halifax, NS, Canada; University of Alberta Faculty of Medicine & Dentistry, Edmonton, AB, Canada; Gastrointestinal Oncology, Endoscopy and Surgery (GOES) research group, Althaia Xarxa Assistencial Universitària de Manresa, Institut de Recerca i Innovació en Ciències de la Vida i de la Salut de la Catalunya Central (IRIS- CC), Manresa, Spain; Gastrointestinal Oncology, Endoscopy and Surgery (GOES) research group, Althaia Xarxa Assistencial Universitària de Manresa, Institut de Recerca i Innovació en Ciències de la Vida i de la Salut de la Catalunya Central (IRIS- CC), Manresa, Spain; Department of Gastroenterology, Hospital Universitario de La Princesa, Instituto de Investigación Sanitaria Princesa (IIS-Princesa), Universidad Autónoma de Madrid (UAM), and Centro de Investigación Biomédica en Red de Enfermedades Hepáticas y Digestivas (CIBERehd), Madrid, Spain; Department of Gastroenterology, Hospital Clínic de Barcelona, Centro de Investigación Biomédica en Red de Enfermedades Hepáticas y Digestivas (CIBERehd), Institut d’Investigacions Biomèdiques August Pi Sunyer (IDIBAPS), University of Barcelona, Barcelona, Spain; Department of Gastroenterology, Hospital Universitario de La Princesa, Instituto de Investigación Sanitaria Princesa (IIS-Princesa), Universidad Autónoma de Madrid (UAM), and Centro de Investigación Biomédica en Red de Enfermedades Hepáticas y Digestivas (CIBERehd), Madrid, Spain; University of Alberta Faculty of Medicine & Dentistry, Edmonton, AB, Canada; Department of Gastroenterology, Hospital Universitario de La Princesa, Instituto de Investigación Sanitaria Princesa (IIS-Princesa), Universidad Autónoma de Madrid (UAM), and Centro de Investigación Biomédica en Red de Enfermedades Hepáticas y Digestivas (CIBERehd), Madrid, Spain

## Abstract

**Background:**

It is still unclear what the optimal treatment regimen for *Helicobacter. pylori* (*Hp*) infection is. The Maastricht VI (2022) and ACG (2024) guidelines recommend bismuth-based quadruple therapy (proton pump inhibitor [PPI], bismuth-metronidazole-tetracycline [PBMT]) for 14 days as first-line therapy for *Hp*. PPI-amoxicillin-metronidazole-clarithromycin (PAMC) is also recommended as first-line therapy by the Toronto Consensus (2016) and Maastricht VI guidelines. PPI-amoxicillin-levofloxacin (PAL) and PPI-amoxicillin-rifabutin (PAR) are accepted rescue therapies for treatment failures.

**Aims:**

To describe the success and adverse event rates of currently prescribed treatments for *Hp* in Canada.

**Methods:**

Multicentre, prospective registry evaluating the decisions and outcomes of *Hp* management by Canadian gastroenterologists (Hp-CanadaReg, WorldHpReg’s partner). Local research ethics board approval was obtained at each recruiting site, and informed consent was obtained from all participants. Data were registered at AEG-REDCap e-CRF. Both treatment naïve and previously treated patients were eligible for the study. Treatment adherence and adverse events were recorded via telephone or in person interviews. A modified intention-to-treat (mITT) analysis was used, which includes all patients with at least one follow-up test-of-cure.

**Results:**

Between August 2024 - October 2025, 112 patients were enrolled across Canada. The majority of patients were treatment naïve (85/112) and 81% (60/74) were cured (Table 1).

For first-line treatments, 91% (39/43) of patients were cured with PAMC vs 100% (5/5) with PBMT. Across all lines, PAL was successful in 50% of patients (1/2), while PAR was successful in 50% (2/4). Among patients treated with PPI-amoxicillin-clarithromycin (PAC, n = 5), 67% were cured (2/3).

At least one adverse event was experienced by 59% (52/88) of patients, including diarrhea (31%, 27/88), nausea (19%, 17/88), dysgeusia (15%, 13/88), and vomiting (11%, 10/88). Adverse events were reported by 57% (31/54) of PAMC- vs 68% (13/19) of PBMT-treated patients (p = 0.398). One serious adverse event occurred in a PAMC patient requiring hospitalization for complications secondary to suspected penicillin allergy.

Most patients (95%, 106/112) received treatment for 14 days and 94% (73/78) completed treatment, taking >90% of the prescribed therapy.

**Conclusions:**

These are the first results from patients enrolled in the prospective Canadian subcohort of the WorldHpReg. The two first-line quadruple therapies, PAMC and PBMT, achieved success rates above >90%. These data provide valuable insight into current *Hp* treatment practices across Canada and will help guide future efforts to optimize the effectiveness of different therapeutic first-line regimens.

**Funding Agencies:**

None